# The Preserving of Food

**Published:** 1919-08-09

**Authors:** 


### THE PRESERVING OF FOOD.

### A Sketch of Some Objects in Research.

In a recent issue (June 28, p. 314) attention was drawn to our needs, both recent and future, of very carefully and critically examining the quality and quantity of the food supplies of this country and to the requirement that the electorate must insist, though the war is now over, that the professional politician continues to take a live and active interest in these problems. The Report of the Food Investigation Board, which was then mentioned, reveals activities and results which have, for the various committees, earned every right of whole-hearted support in their work by every person who has the country's welfare truly at heart. Below it is attempted to outline the bearings which these researches will have on the food problems of the future, and to indicate briefly some of the scientific questions which must be dealt with.

As the field to be covered by the work of the Board is very extensive, it was decided to divide it among several committees, the members of which have expert knowledge of the subject assigned to them. These are (1) Fish Preservation Committee; (2) Engineering Committee; (3) Meat Preservation Committee; (4) Fruit and Vegetables Committee; (5) Oils and Fats Committee; (6) Canned Foods Committee. Most have appointed sub-committees dealing with special branches of their work. Special laboratories have been started and experimental plants erected at Billingsgate, Cambridge, Lowestoft, and other places, and some of the results are already published in the report for 1918.

### The Preserving of Fish.

The Fish Preservation Committee has been mainly occupied in investigating "brine freezing," in which the fish to be frozen are plunged into strong brine cooled to 10°F. or lower. Owing to the high specific heat of the brine cooling is very rapid, and the separation of fluid and solid which occurs in the ordinary slow process of freezing is prevented. The result is that the flesh remains fresh when thawed and the colour and superficial appearance are preserved. It is hoped by this process it will be possible to preserve gluts of fish which have hitherto been destroyed for want of some reliable method of preservation.

### Frozen Meat Supplies.

There is much work to be done by the Meat Committee—for instance, while mutton can be easily frozen without damaging it as food, beef requires much more careful handling. It is stated "freezing in the ordinary way, that is by cold air, causes a separation of fluid in the substance of the muscle fibres, with the result that on thawing, unless somewhat elaborate precautions are taken, there is loss of water and soluble constituents, and the texture of the meat is impaired." The cause of this sensitiveness is unknown and raises several questions. Is it due to abstracting heat from so large an animal as the ox, or to the "colloidal properties and chemical constitution of the muscle fibres themselves"? Or, "is the permeability of the sarcolemma of the muscle fibre of the ox different from that of the sheep"? These are obviously very difficult questions and can only be settled by proper scientific investigations. This work is to be supervised by Professor W. H. Bayliss. Other important questions also require investigation, such as autolysis, and the chemistry of the flavouring substances in meat. In connection with autolysis, the work so far done is sketched as follows: "The organisms found in meat in the earlier stages of putrefaction are being isolated in pure culture and their growth followed." Further, "in order to establish the nature of their action it is necessary to discover some method of sterilising meat which shall not so change its physical and chemical character as to render the results of experiments incapable of application to the actual conditions existing in the industry in which the organisms have to make good their footing and grow in flesh which has been subject only to intrinsic *post-mortem* change. Sterilisation by heat is obviously inapplicable." There is also to be a systematic investigation into the moulds found in cold stores and this will be most welcome to sanitary officers who have to examine frozen carcases of beef and mutton—for it is one of the greatest difficulties to know what to reject and what to pass when a whole consignment from a ship becomes more or less infected by various moulds, many of which are comparatively harmless, though they disfigure a carcase and to the inexperienced eye render it unfit for consumption.

### Edible Oils and Fats.

The Oils and Fats Committee have a hardly less onerous task before them than the foregoing. Their general reference is "a survey of the sources of supply of edible oils and fats with the object of increasing the proportion available for human consumption." This is a very general reference, but included in it will be the important question of vitamines in oils and the nutrient qualities in this respect of vegetable and animal oils. This latter, one might say, is extremely important, especially as margarine is one of the staple fats of the poor and there has been a tendency to make this almost entirely from vegetable fats, most of which are extremely poor in vitamine.

The following are the terms of reference of the Fruit and Vegetables Committee: To consider and report on

1. The metabolism of fruit and vegetables at low temperatures; (2) the use of cold, of gases, and of desiccation for the preservation of fruit and vegetables; and (3) a conspectus of such diseases of fruit and vegetables as are of importance in connection with their keeping properties.

**The Preserving of Food**—(*continued*).

This is a good indication of the scope of inquiry, for as the report points out, fruit and vegetables are living structures which are killed by being frozen. Experiments must therefore be conducted at or about freezing point, and a thermostat, capable of maintaining suitable temperature for long periods, will have to be invented. There are also many technical difficulties in connection with the obtaining and preserving of fruit and vegetable juices for chemical analysis.

Tinned Food Problems.

The Canned Food Committee have also much important work, and nothing would be more gratifying to sanitary officers than a standardisation of the methods of examining canned foods, especially meat, and meat products, for owing to new methods of canning, the old criteria by which tins were passed or rejected have become more or less useless. For instance, if tins of canned meats showed any slackness or could be indented at either end by pressure of the fingers, or gave forth a hollow sound, they were at once rejected, since these signs were taken to indicate that gas formation was proceeding from putrefaction of the contents. Now, however, canning is done in a vacuum chamber, and atmospheric air which was formerly completely expelled by heat, may be left behind, especially if the vacuum is incomplete, thus giving the sealed tin the appearance of being partially "blown," while the contents on opening may be found to be perfectly sound.

It is to be hoped that the Food Investigation Board will not confine their attentions to the subject-matter indicated in the report, for there are many other foods which are preserved in cold store and require most careful handling not mentioned, *e.g.*, eggs, milk, and cream, cheese, game, fowl, oysters, and cockles, "offal," beverages, etc. Their present choice of subjects is no doubt made on account of the articles being in common use and staple foods, for when the best condition of preserving them has been thoroughly gone into, it will be easy to extend the inquiries into all kinds of food.

